# Anomalous Extensor Digiti Minimi with Multiple Slips and Bulbous Appearance

**DOI:** 10.1016/j.jhsg.2023.04.010

**Published:** 2023-05-18

**Authors:** Mario Giacobazzi, Neil Singh

**Affiliations:** ∗ Lake Erie College of Osteopathic Medicine, Erie, PA; † UPMC Jameson, New Castle, PA

**Keywords:** Anomalous, Bulbous, Extensor digiti minimi, Multislips, Tenosynovitis

## Abstract

The extensor digiti minimi is a muscle in the posterior compartment of the forearm that extends the fifth digit. Variations of the extensor tendons of the hand are common and mostly asymptomatic, however, some may impinge and occupy the narrow dorsal compartments of the wrist causing dorsal wrist pain and impairment of digital movement. Orthopedic literature illustrates how frequent anomalies of the extensor indicis proprius, extensor digitorum brevis manus, and extensor medii proprius occur; however, minimal literature documents a bulbous, multi slip extensor digiti minimi. Within this case, a 30-year-old, right-handed woman with no prior hand trauma presented with recurrent snapping localized to her right fifth digit, causing intermittent pain and an audible “click”. This study aims to provide a thorough anatomical description of a rare extensor digit minimi anomaly and a viable option to treat successfully an inflamed, symptomatic extensor retinaculum affecting the extensor digiti minimi.

Anatomical variations in the hands are common. Embryologic maturation of the precursor extensor muscle mass has an important role in anomaly development. The precursor extensor muscle mass develops into three separate parts: radial, superficial, and deep. The greatest variation occurs in the deep portion, which goes on to form the abductor pollicis longus, extensor pollicis brevis on the radial side, extensor pollicis longus on ulnar side, and extensor indicis proprius on ulnar side.^1^ The superficial portion forms the extensor digitorum communis, extensor carpi ulnaris, and extensor digiti minimi. A prior case report conducted by Sasaki et al^2^ concluded that >58% of cadavers in their study had duplications of the extensor digiti minimi.

Dorsal compartments in the hand tend to be relatively crowded. Anomalous muscles or synovitis further increase the congestion and may result in pain if inflamed or irritated. The extensor digiti minimi tendon passes under the extensor retinaculum near the wrist and then passes through the fifth extensor compartment of the wrist and ends at the base of the fifth digit. The pathway of the tendon through the extensor retinaculum enables possibility of impingement with any anatomic differentiations.

## Case Report

Written informed consent was obtained from the patient for publication of this case report and accompanying images. A 30-year-old right-handed woman presented with the chief complaint of fifth-digit pain and a prominent lump on her right wrist. She denied any specific injury connected with onset of symptoms and endorsed a 6-month history of pain and symptomatology. The patient explained that sharp pain occurred with immediate onset following extension of the fifth digit. Overall, the pain was stable with intermittent achiness throughout the day. She stated that she had been sleeping with a splint to help minimize movement throughout the night. Splinting, along with rest, seemed to help minimize pain as the patient denied waking up at night from pain. At the time of the visit, she was experiencing major swelling which caused considerable patient discomfort. She mentioned that her small finger felt as though it was snapping over the dorsal aspect of the fifth metacarpal.

A few weeks before presenting to our clinic, she underwent magnetic resonance imaging of the right wrist, which showed prominent thickening of the extensor digiti minimi tendon within the wrist extensor region localized at the level of the distal ulnar styloid. Initial plain films obtained at this visit demonstrated a possible marginal osteophyte at the base of the fifth metacarpal on the right wrist when compared to the left wrist. The films were unremarkable for any acute osseous abnormalities.

Examination of the upper extremities revealed warm, well-perfused hands bilaterally, with no visible rashes or lesions noted, and normal skin temperature and turgor. The patient presented with a swollen and tender mass at the base of the dorsal right fifth metacarpal. Her extensor carpi ulnaris was stable. She was nontender to palpation over the ulnar styloid. An acute pain test conducted at the distal radial ulnar joint was negative. She was able to make a fist with both hands and full range of motion remained intact. Her strength was normal with no gross instability. There was palpable swelling at the base of the fifth metacarpal with a reproducible snapping sound and sensation (Video 1; available on the *Journal’*s website at www.jhsgo.org). Initial diagnosis was right extensor digiti minimi tenosynovitis with snapping of the small finger.

The nature of the condition was discussed with the patient at this time regarding her tenosynovitis as well as snapping of the fifth digit. Various treatment options were discussed, including conservative care with bracing, oral or topical anti-inflammatory agents, and potential cortisone injections. Because of the considerable mechanical symptoms experienced by the patient, she chose a more definitive solution, tenosynovectomy under local anesthesia, which would preserve her ability to extend the fifth digit and aid in identification of the snapping. The planned procedure included debridement, extensor tenosynovectomy, release of the extensor digiti minimi tendon and retinaculum, and possible excision of the marginal osteophyte. Before surgery, an ultrasound was conducted to visualize the extensor digiti minimi tendon (Video 2; available on the *Journal’*s website at www.jhsgo.org). The patient was instructed to flex and extend her right fingers while imaging was recording.

A standard longitudinal approach was used for surgical exploration. The initial incision was aimed at the top of the extensor retinaculum at the level of the visible mass. A longitudinal incision followed. The extensor retinaculum was identified at the level of the extensor digitorum minimi tendon. At this location, there was a distal thickening and inflammation. The patient moved the digit through an active range of motion. The bulbous snapping tendon was identified, and the snapping occurred as it traversed beneath the accessory sheath (Video 3; available on the *Journal’*s website at www.jhsgo.org).

**Figure 1:**
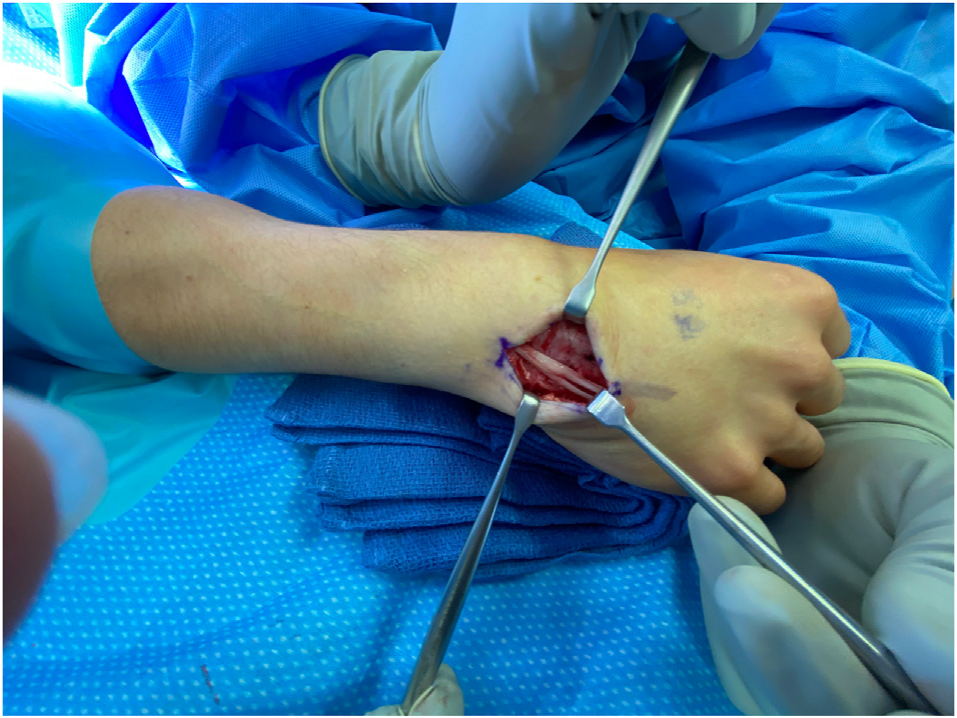
Anomalous EDM with multiple slips.

The retinaculum was incised carefully because of thickening and inflammation. At that point, the anomalous extensor digiti minimi with multiple slips was well visualized. The tendon showed a bulbous appearance and there was considerable fluid retention and inflammation above the tendon (Video 4, available on the *Journal’*s website at www.jhsgo.org; Fig. 1). Tenosynovectomy was performed carefully, which aided in exposure of the tendon and retinaculum.

Due to the bulbous nature of the tendon and thickening of the retinaculum, we decided to excise the retinaculum to prevent risk of continued irritation of the tendons. The distal portion of the retinacular sheath then was repaired. Again, the patient took her digits through an active range of motion, which revealed that the snapping of the tendon had resolved and the anomalous tendon was gliding smoothly (Video 5; available on the *Journal’*s website at www.jhsgo.org).

The patient followed 2 weeks after surgery in an outpatient clinic and she continued to be pleased with the progress. After surgery, the patient denied any reproducible clicking or instability. Physical examination showed that the extensor tendons remained centralized through complete range of motion. There was no active clicking of the fifth digit. No extensor lag was noted. She gradually returned to activities and continued at home exercises along with hand therapy.

Two months after surgery, the patient reported neither pain nor swelling. Evaluation of the right hand showed intact full range of motion. No clicking or snapping was noted. She had a grossly intact neurological examination. Her digital snapping had resolved completely.

## Discussion

The literature highlights the commonality of anomalous extensor muscles in the hand. A thorough literature review concluded that the extensor medii proprius was the most common variation, followed by the extensor indicis radialis.^3^^.^ Two variations are located on the more radial aspect of the dorsal hand. An anomalous extensor indicis proprius presents similar to an anomalous extensor digiti minimi. However, an anomalous extensor indicis proprius is more localized within the fourth dorsal compartment as that is where the main muscle belly extends beyond.^1^

A prior case report by Baker and Gonzalez^4^ detailed a snapping wrist similar to what presented in our patient. In this case, the patient has an anomalous extensor indicis proprius. Surgical exploration revealed a disproportionately large muscle belly located under the extensor retinaculum. The large muscle belly caused subluxation of the fourth- and fifth-digit tendons creating the snapping noise.

Sasaki et al^2^ presented a case report featuring a radial bifurcation of the extensor digiti minimi tendon, resulting in pain. Through surgical exploration, a small radial slip was observed, and the bifurcation was noted under the distal portion of the extensor retinaculum. The patient was treated with a surgical release of the extensor retinaculum and resection of the small radial slip. Stenosing tenosynovitis was most likely the key contributing factor to the wrist pain.

Anatomical variations of the extensor digiti minimi (EDM) are less common than other extensor variations. A study analyzing cadaver anatomic trends concluded that the EDM tendon split distal to the extensor retinaculum 75%.^1^ Another study found that 71% of the EDM tendons had two slips and 23% had three slips. Further evaluation resulted in discovery of 73% of EDM tendons possessing accessory retinacular bands surrounding them.^5^ A literature review found a duplication of EDM in 7/12 cases.^2^

The best treatment method to relieve pain caused by tendon impingement seemed to be resection of the extensor retinaculum. Athletes exhibiting dorsal wrist pain from repetitive hyperextension movements had extensor retinaculum impingement in many cases. Five of 7 athletes in one series decided to undergo surgery to resect the extensor retinaculum and they all experienced complete relief of pain.^6^

Variations in the anatomical structure of the EDM tend to be rarer compared to other extensor tendon variations in the hand. Most EDM tendons showed bifurcation distal to the EDM and only a few showed three slips. The bulbous appearance seen in our patient caused stenosing tenosynovitis, which resolved after surgical release. Excision of the extensor retinaculum proved to be the best treatment method for long term symptom relief. After surgery, our patient experienced a remarkable reduction in pain and improvement in her overall quality of life.

## Supplementary Data

Video 1 Patient undergoes active flexion and extension of the MCP joints in her right hand which produces an audible snapping of extensor digiti minimi.

Video 2 Ultrasound of extensor digitorum minimi through active flexion and extension. Snapping of the tendon is visualized in this film study.

Video 3 Bolbous snapping tendon throughout active movement underneath the extensor retinaculum.

Video 4 Anomalous extensor digiti minimi with multiple slips following incision of the ER. The film shows the tendon slips through active range of motion.

Video 5 Anomalous extensor digiti minimi motion following removal of sheath.
